# Contraceptive method choice among women in slum and non-slum communities in Nairobi, Kenya

**DOI:** 10.1186/s12905-016-0314-6

**Published:** 2016-07-12

**Authors:** Rhoune Ochako, Chimaraoke Izugbara, Jerry Okal, Ian Askew, Marleen Temmerman

**Affiliations:** Faculty of Medicine and Health Sciences, Ghent University, Ghent, Belgium; African Population and Health Research Center, Nairobi, Kenya; Population Council, Nairobi, Kenya; World Health Organization, Geneva, Switzerland; International Centre for Reproductive Health, Ghent University, Ghent, Belgium

**Keywords:** Contraceptive method choice, Contraceptive use, Slum, Non-slum, Urban poor, Nairobi, Kenya

## Abstract

**Background:**

Understanding women’s contraceptive method choices is key to enhancing family planning services provision and programming. Currently however, very little research has addressed inter and intra-regional disparities in women’s contraceptive method choice. Using data from slum and non-slum contexts in Nairobi, Kenya, the current study investigates the prevalence of and factors associated with contraceptive method choice among women.

**Methods:**

Data were from a cross-sectional quantitative study conducted among a random sample of 1,873 women (aged 15–49 years) in two non-slum and two slum settlement areas in Nairobi, Kenya. The study locations were purposively sampled by virtue of being part of the Nairobi Urban Health and Demographic Surveillance System. Bivariate and multivariate logistic regression were used to explore the association between the outcome variable, contraceptive method choice, and explanatory variables.

**Results:**

The prevalence of contraceptive method choice was relatively similar across slum and non-slum settlements. 34.3 % of women in slum communities and 28.1 % of women in non-slum communities reported using short-term methods. Slightly more women living in the non-slum settlements reported use of long-term methods, 9.2 %, compared to 3.6 % in slum communities. Older women were less likely to use short-term methods than their younger counterparts but more likely to use long-term methods. Currently married women were more likely than never married women to use short-term and long-term methods. Compared to those with no children, women with three or more children were more likely to report using long term methods. Women working outside the home or those in formal employment also used modern methods of contraception more than those in self-employment or unemployed.

**Conclusion:**

Use of short-term and long-term methods is generally low among women living in slum and non-slum contexts in Nairobi. Investments in increasing women’s access to various contraceptive options are urgently needed to help increase contraceptive prevalence rate. Thus, interventions that focus on more disadvantaged segments of the population will accelerate contraceptive uptake and improve maternal and child health in Kenya.

## Background

Globally, 600,000 women die annually due to pregnancy-related causes, and 75,000 die as a result of unsafe abortions with 99 % of these deaths occurring in developing countries [1–3]. Failure or lack of contraceptive services is the cause of about 200,000 of these maternal deaths. Women who have unintended births tend to suffer postpartum depression, feelings of powerlessness, increased time pressures, and a reduction in overall physical health [4, 5]. They also have poorer quality relationships with their children which potentially can lead to physical abuse and less attention [6, 7]. Often, children from large families compete for scarce family resources which likely leads to overall poor quality of life. To address some of these challenges, a study done by the Population Action International has shown that infant mortality in developing countries could be decreased by one-third by increasing the spacing between births to 2–4 years [8]. In all, effective use of contraception results in healthy and socially beneficial life for mothers, their children and households [9]. Moreover, it has been proven that contraceptive use prevents unintended pregnancies and abortions and facilitates family planning and spacing of births. Furthermore, effective contraception improves the social and economic role of women and enables them to participate fully in society [3]. These benefits of family planning remain central in achieving Millennium Development Goals (MDGs) target of attaining universal access to reproductive health and sustainable millennium development goals beyond 2015 [10, 11].

Contraceptive method choice is an indication of existing quality of care for women. A wide range of contraceptive options is a sign that programs can meet the diverse needs of women [12]. Availability of both short-term and long-term methods ensures that the specific needs of women who intend to limit family size, space and delay births are met and their concerns about sexually transmitted infections (STIs) and cultural acceptability of available methods is within their reach [13]. The landmark International Conference on Population and Development (ICPD) of 1994 called for greater recognition of complexities and differences in the family planning needs and preferences of couples and individuals. Hence it is imperative that both women and men have access to information and a wide range of safe and effective family planning methods that will enable them exercise freedom of choice [14]. Existing evidence indicates that restricted contraceptive choice often leads poor uptake and low contraceptive prevalence [14]. Over the years, contraceptive prevalence rates has grown exponentially in Kenya from 9.7 % in 1984 to 46 % in 2008-09 and recently to 58 % in 2014 among married women [15-17]. However, unmet need for family planning and unintended pregnancy remain persistently high, suggesting underlying barriers to effective contraception. According to the 2008–09 Kenya Demographic and Health Survey (DHS), 42 % of married women described their current pregnancy as unintended [15]. The 2014 Kenya DHS reports that unmet need for family planning is 18 % among married women [17].

Most studies around contraceptive use have primarily been informed by national demographic surveys that portray data aggregated at national or regional level thereby leaving gaps in explaining inter and intra-regional disparities [15]. For instance, increased urbanization in Kenya has led to calls for more accessible family planning services in urban areas. Although it is assumed that urban residents have better access to health services than their rural counterparts, existing evidence suggests that this might not be true given the varied living conditions found in cities. Specifically, urban residents living in informal or slum settlements face several socio-economic and health challenges. In Nairobi, it is estimated that 60 % of the population are living in slums [18, 19]. Mostly, slum settlements are characterized by high poverty levels, poor infrastructure, inadequate access to water and sanitation facilities and lack of basic amenities. Slum dwellers face other challenges such as high levels of unemployment, crime, substance abuse, poor schooling facilities and early sexual debut and low use of contraceptives which is directly or indirectly connected to unplanned childbearing [20]. Generally the use of contraceptives among the urban poor remains low [21]. Urban poor families are also often larger than their wealthier counterparts. This may suggest a lack of access to family planning for spacing and a wide range of options to limit births [22].

This study therefore seeks to understand contraceptive use and specifically the choice between no method, traditional, short-term and long-term methods among women living in slum and non-slum contexts in Nairobi. An understanding of the socio-economic and demographic drivers of women’s contraceptive use can serve as efforts to improve the uptake of family planning services and interventions. It is hypothesized that women residents in slums may not have a wide range of family planning options compared to their non-slum counterparts thereby limiting their choice of short-term verses long-term methods and use of modern contraceptives in general. Specifically, the study seeks to address the following objectives: a) determine the prevalence of contraceptive method choice by characteristics of the study population; b) explore the association between contraceptive method choice; and c) identify socio-demographic, socio-economic and behavioural/attitudinal determinants of contraceptive method choice among women from slum and non-slum settlements.

## Methods

### Study setting

The larger study, focused on women living in two non-slum settings (Harambee and Jericho) and two slum settlements (Korogocho and Viwandani) in Nairobi, Kenya. The settlements were purposively selected by virtue of being part of the Nairobi Urban Health and Demographic Surveillance System (NUHDSS), a research platform of the African Population and Health Research Center (APHRC) [23]. All the four settlements are also recognized as distinct communities and have chiefs appointed by the government of Kenya. Though their residents are socially and economically heterogeneous, Korogocho and Viwandani are densely populated settlements occupied largely by economically disadvantaged people. The two settlements are also characterized by high unemployment and poverty levels, crime, poor sanitation and high prevalence of risky sexual behaviors and poor sexual and reproductive health outcomes, compared to Nairobi as a whole [24–26]. Health and other facilities in Korogocho and Viwandani are very poorly resourced and often lack basic essentials. Poverty also prevents a large number of people in both settlements from accessing better quality services in the city. Viwandani is located in Nairobi East District occupying an area measuring 5.7 km^2^. Viwandani has a total of 17,926 households [26, 27]. It is located within the industrial area part of Nairobi, about 7 km from Nairobi city center. The informal settlement is characterized by overcrowding, insecurity, poor housing and sanitary conditions, and inadequate social amenities [26, 28]. Korogocho is in Nairobi North District occupying an area of 0.9 km^2^, located within Kasarani Division. It is situated approximately 11 km from Nairobi’s central business district. The informal settlement has a total of 12,909 households [27]. Most residents operate small businesses to earn their living as wage employment is difficult to come by. The slum is characterized by high levels of insecurity, poor accessibility, inadequate housing, poor sanitation and water quality, and low access to basic services like health care and education. Jericho and Harambee, are also characterized by socio-economic diversity, but unlike the slums communities are predominantly middle-class settings, and enjoy better health, access to quality to services, and other indicators [29–31]. They were established during the pre-colonial period as predominantly African settlements. They have relatively better residential structures including accessible feeder roads, drainage and sewerage system [32].

### Source of data

This paper uses data from a cross-sectional quantitative research project conducted in 2009/10 in two non-slum settings (Harambee and Jericho) and two slum settlements (Korogocho and Viwandani) in Nairobi, Kenya. While these communities are not contiguous, they, form the Nairobi Urban Health and Demographic Surveillance System (NUHDSS), a research platform of the African Population and Health Research Center (APHRC). All four settlements are also recognized as distinct communities and have chiefs appointed by the government of Kenya. Though their residents are socially and economically heterogeneous, Korogocho and Viwandani are densely populated settlements occupied largely by economically disadvantaged people. The two settlements are also characterized by high unemployment and poverty levels, crime, poor sanitation and high prevalence of risky sexual behaviors and poor sexual and reproductive health outcomes, compared to Nairobi as a whole [18, 19]. Health and other facilities in Korogocho and Viwandani are very poorly resourced and often lack basic essentials. Poverty also prevents a large number of people in both settlements from accessing better quality services in the city [20]. Jericho and Harambee are also characterized by socio-economic diversity, but unlike the slums communities studies are predominantly middle-class settings, and enjoy better health, access to quality to services, and other indicators [21–23]. The study was based on a sample of randomly-selected women aged 15–49 years, using a two-stage sampling procedure. In the first stage, 1,000 households from the two slum settlements and 1,000 households from the two non-slum settings were drawn from the NUHDSS. A second stage consisted of a random selection of one eligible woman (usual resident aged 15–49 years) in each of the sampled households [30, 31]. The sample size was based on the practice by the demographic and health surveys (DHS), which typically assume that to obtain reasonable precision for most indicators, at least 800 completed interviews of women 15–49 years are needed in each domain. Accounting for possible missing data and non-responses, the sample size was set to 1,000 per area. The questionnaire sought information on respondents’ social, economic, demographic, pregnancy and birth histories (including miscarriages and abortions, stillbirths, and neonatal deaths), the intendedness of all pregnancies mentioned by the respondent irrespective of their outcomes, current use of contraception and specific methods used. A total of 1,962 women were successfully interviewed, yielding a response rate of 98.1 %. This paper analyses data from 1873 women who reported being sexually active. We exclude from our analysis, 89 women who reported that they had never had sex or were pregnant at the time of the survey.

### Study variables

The question that reported current contraceptive use among women was as follows: ‘Are you CURRENTLY doing anything to avoid getting pregnant?’ those who responded with a ‘*yes’* were further asked to state the method they were currently using. The options listed included: female sterilization, male sterilization, pill, IUD (e.g., coil), injectables (e.g., Depo), implants, male condoms, female condoms, lactational amenorrhea method (LAM), rhythm method (safe days), withdrawal, emergency contraception (e.g., e-pill), diaphragm, spermicide (e.g., gel, form), and other methods not listed above for which they were required to specify. From these categories, the outcome variable, contraceptive method choice, was measured as a four outcome variable coded as: ‘*no method*’ for women who reported not doing anything to prevent pregnancy, ‘*traditional method*’ for women using withdrawal and the rhythm methods which are less effective in pregnancy prevention; *short*-*term methods* (for women who reported using female and male condoms, injectables, pills, emergency contraception); and *long*-*term methods* (for women who reported using female and male sterilization, implants and IUD). The dependent variable, household wealth was computed from reported household possessions, amenities and dwelling characteristics using principal component analysis and recoded into tertiles; poor, medium, and rich [33, 34]. Measurement of pregnancy wantedness is based on questions about the desirability of recent pregnancies reported. The question asked to women was as follows *“At the time you became pregnant with (NAME), did you want to become pregnant****then****, did you want to wait until****later****, or did you****not want****to have another (more) children at all?”*, the response was classified into three categories; never pregnant, intended pregnancy (for women who reported they wanted the pregnancy at the time of conception), and unintended (for women who reported wanting no more children and wanting later the pregnancy later than at the time of conception). Employment status was defined as self-employed for those who were engaged in their own means of earning income, informal employment referred to those engaged in income that are partially or fully outside government regulation, formal employment were those under government taxation regulation while the unemployed were those not engaged in any income generating activities.

Contraceptive method choice is influenced by several factors. In this study, we hypothesize that three sets of factors, socio-demographic, socio-economic and behavioural/attitudinal factors as the major influencers of contraceptive method choice. Socio-demographic factors include age, marital status, ethnicity, parity, and household size. The level of education, wealth, type of residence and employment status are considered as socio-economic factors. Pregnancy wantedness on the other hand is considered as a behavioural/attitudinal factor. This conceptual framework makes an assumption that all these factors directly influence the choice a woman makes on the contraceptive method. Level of education is coded as none, primary and secondary/higher while wealth index is recoded as tertiles and labelled poor, middle and rich.

### Methods of analysis

Using statistical software STATA version 14 for the analysis, descriptive statistics were used to provide sample characteristics. Secondly, bivariate analysis was used to assess individual relationship of each explanatory variable with contraceptive method choice while multivariate analysis was used to assess relationships controlling for other explanatory variables. The dependent variable, a four outcome variable coded as no method, traditional methods, short-term and long-term methods was fitted in a multinomial model to predict the determinants of contraceptive method choice among women living in slum and non-slum settlements. Three models were fitted, Model I assessed the determinants of contraceptive method choice while controlling for socio-demographic factors, Model II controlled for socio-economic factors while model III controlled for behavioural/attitudinal factor. The results of the regression analyses have been presented by odds ratio (OR) with 95 % confidence interval. All analyses were weighted using the svy command to account for differences in sampling probabilities.

## Results

### Sample characteristics

Table 1 presents results from 1873 women, 28.2 % reported use of no method, 34.2 % were using traditional methods while 31.2 % and 6.4 % were using short-term and long-term methods respectively. Majority of the women interviewed were aged 15–24 years while 43.3 % were currently married. Considering the women by their ethnic groups, Kikuyu women 33.1 %, were the majority, women who reported having 1–2 children were 38.3 % while about half of the households, 48.9 %, had between 4–6 members. Majority of the women had no education, 40.1 %, and as expected, wealth was almost equally split among the four categories. There were slightly more women living in the non-slum settlements, 50.6 %. Considering employment status, about half, 47.5 %, of the women were unemployed and another 23.6 % being self-employed. About half, 48.8 %, of the women reported that their pregnancy was intended.

**Table 1:** Sample characteristics of women 15–49 years living in slum and non-slum settlements in Nairobi, Kenya

Characteristics	Percent (%)	Number
Contraceptive method choice		
No method	28.2	528
Traditional method	34.2	641
Short-term method	31.2	584
Long-term method	6.4	120
Socio-demographic factors		
*Age*		
15–24	36.0	675
25–34	35.9	673
35–54	28.0	525
*Marital status*		
Never married	40.6	761
Currently married	43.3	811
Formerly married	16.1	301
*Ethnicity*		
Kikuyu	33.1	620
Luhya	18.1	339
Luo	18.7	351
Kamba	17.6	329
Other	12.5	234
*Parity*		
No children	31.5	590
1–2 children	38.3	718
3+ years	30.2	565
*Household size*		
1–3 members	24.0	448
4–6 members	48.9	915
7+ members	27.1	507
Socio-economic factors		
*Education*		
None	40.1	751
Primary	35.5	664
Secondary/higher	24.5	458
*Wealth index*		
Poor	34.1	638
Medium	32.9	617
Rich	33.0	618
*Residence*		
Slum	49.4	926
Non-slum	50.6	947
*Employment status*		
Self-employed	23.6	441
Informal	10.8	203
Formal	18.2	340
Unemployed	47.5	889
Behavioral/attitudinal factors		
*Pregnancy wantedness*		
Never pregnant	31.5	590
Intended	48.8	914
Unintended	19.7	369
Total (N)	100.0	1873

### Contraceptive method choice and settlement type

**Fig. 1:**
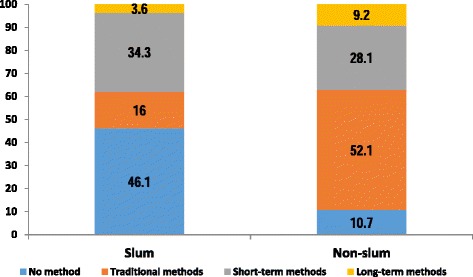
Bivariate association between residence and contraceptive method choice

Figure 1 shows contraceptive method choice by type of urban residence. Reported use of no family planning was high in the slum settlements while for the non-slum settlements, use of traditional methods was slightly more than half (52.1 %) among the women. Women living in the non-slum settlements reported a slightly higher use of long-term methods, 9.2 % compared to 3.6 % among women living in the slum settlements. These results are as presented in Fig. 1.

### Prevalence of contraceptive method choice among women living in slum and non-slum settlements

Table 2 shows the prevalence of contraceptive method choice in relation to selected factors including socio-demographic status, socio-economic status, and behavioural/ attitudinal among sexually active women. Considering the socio-demographic characteristics, there exists a significant positive association between age and use of traditional methods. Women over 25 years were less likely to use a traditional method as compared to using no method. Similarly, women aged 35 years and above were less likely to use a short-term method than no method, compared to younger women of aged 15–24 years. On the other hand, women aged 25 years and above were more likely to use a long-term method than use no method compared to those aged under 25 years. Currently and formerly married women were less likely (*p* < 0.001) to use a traditional method than use no method compared to their never married counterparts. On the contrary, currently married women were more likely to use a short-term method than use no method compared to never married women. Both currently and formerly married women were more likely to use a long-term method than use no method compared to their never married counterparts. Considering ethnic affiliation, Luo women were more likely (*p* < 0.05) to use a traditional method than use no method compared to Kikuyu women, on the other hand, Kamba women were less likely to use a long-term method compared to Kikuyu women. Women with at least one child were less likely to report use a traditional method, but more likely to report use of a short-term or long-term method compared to those who had no children. Households with at least 4 members were more likely to report use of a traditional, short-term or long-term method compared to those with 1–3 members.

**Table 2:** Association between contraceptive method choice and various background characteritics of women aged 15–49 years

Characteristics	Traditional method vs. no method			Short-term method vs. no method			Long-term method vs. no method		
Socio-demographic factors									
Age									
15–24	1.00			1.00			1.00		
25–34	0.44	***	[0.31–0.61]	1.46	*	[1.07–2.01]	2.59	*	[1.15–5.79]
35–54	0.36	***	[0.26–0.51]	0.52	***	[0.36–0.74]	3.02	**	[1.38–6.62]
Marital status									
Never married	1.00			1.00			1.00		
Currently married	0.32	***	[0.23–0.44]	2.11	***	[1.53–2.91]	6.71	***	[2.84–15.84]
Formerly married	0.20	***	[0.13–0.31]	0.92		[0.62–1.37]	3.40	*	[1.27–9.11]
Ethnicity									
Kikuyu	1.00			1.00			1.00		
Luhya	1.43		[0.93–2.20]	1.35		[0.89–2.07]	1.18		[0.56–2.48]
Luo	1.54	*	[1.00–2.35]	1.39		[0.91–2.12]	0.64		[0.28–1.48]
Kamba	0.81		[0.54–1.21]	1.22		[0.85–1.74]	0.39	*	[0.18–0.88]
Other	1.02		[0.67–1.55]	0.78		[0.51–1.19]	0.51		[0.23–1.15]
Parity									
No children	1.00			1.00			1.00		
1–2 children	0.36	***	[0.26–0.51]	2.97	***	[2.03–4.34]	5.74	**	[1.92–17.16]
3+ years	0.20	***	[0.14–0.29]	1.72	**	[1.16–2.54]	7.63	***	[2.60–22.35]
Household size									
1–3 members	1.00			1.00			1.00		
4–6 members	1.96	***	[1.39–2.77]	1.39	*	[1.02–1.88]	3.69	***	[1.77–7.71]
7+ members	2.40	***	[1.63–3.52]	0.93		[0.64–1.35]	2.60	*	[1.11–6.05]
Socio–economic factors									
Education									
None	1.00			1.00			1.00		
Primary	2.42	***	[1.75–3.35]	1.34		[0.99–1.80]	1.23		[0.64–2.35]
Secondary/higher	11.26	***	[7.02–18.07]	3.22	***	[2.00–5.20]	9.30	***	[4.76–18.18]
Wealth index									
Poor	1.00			1.00			1.00		
Medium	1.16		[0.80–1.66]	0.88		[0.63–1.22]	0.92		[0.49–1.73]
Rich	1.44	*	[1.02–2.03]	0.83		[0.60–1.15]	0.73		[0.39–1.36]
Residence									
Slum	1.00			1.00			1.00		
Non-slum	19.95	***	[13.59–29.29]	4.26	***	[2.89–6.28]	13.91	***	[7.90–24.51]
Employment status									
Self-employed	1.00			1.00			1.00		
Informal	1.05		[0.63–1.78]	1.01		[0.65–1.56]	0.82		[0.32–2.07]
Formal	2.54	***	[1.58–4.08]	1.23		[0.78–1.94]	2.75	**	[1.36–5.54]
Unemployed	1.99	***	[1.38–2.86]	1.03		[0.75–1.41]	0.72		[0.37–1.41]
Behavioral/attitudinal factors									
Pregnancy wantedness									
Never pregnant	1.00			1.00			1.00		
Intended	0.25	***	[0.18–0.34]	2.30	***	[1.60–3.30]	6.48	***	[2.24–18.74]
Unintended	0.41	***	[0.27–0.62]	2.49	***	[1.61–3.84]	7.87	***	[2.51–24.70]

^*^ *p* < .05; **p < .01; ***p < .001

Associations with socio-economic factors show that women with secondary and higher education were more likely to use a traditional, short-term or long-term method compared to using no method than those who had no education. Women from rich households were more likely to use a traditional method than use no method compared to those from poor households. Considering type of residence, women living in non-slum settlements were more likely to use traditional, short-term or long-term methods than use no method compared to those living in the slum settlements.

Women in formal employment were more likely to use a traditional or long-term method than use no method compared to those who were self-employed. Whereas behavioural/attitudinal factor, pregnancy wantedness, shows that women with intended and unintended pregnancies were less likely to use a traditional method than use no method, these women were more likely to use a short-term or long-term method than use no method compared to their never pregnant counterparts.

### Determinants of contraceptive method choice among women living in slum and non-slum settlements

Multinomial regression shown on Table 3 was applied using three models to assess the effect of explanatory factors on contraceptive method choice among sexually active women living in slum and non-slum settlements. Model I controlled for the effect of socio-demographic and it shows that women aged 35 years and above were less likely to use a short-term method than use no method. The model further shows that currently married women were more likely to use a short-term or long-term method than use no method compared to their never married counterparts, both currently and formerly married women were less likely to use a traditional method than use no method compared to women who were never married. Considering ethnic affiliation, women from other ethnic groups were less likely to use a short-term method compared to their Kikuyu counterparts, similarly, women from the Kamba community and those from other ethnic communities were less likely to use a long-term method than use no method compared to Kikuyu women. Women with at least one child were more likely to use a short-term method than use no method compared to those who had no children. On the other hand, women who had 3 or more children were less likely to use a traditional method than use no method compared to those with no children. Household size was also an important determinant of contraceptive method choice where households with 4 or more being more likely to use a traditional, short-term or long-term method than use no method compared to those with 1–3 members.

**Table 3:** Determinants of contraceptive method choice among women living in slum and non-slum settlements

Characteristics	Traditional method vs. no method			Short-term method vs. no method			Long-term method vs. no method		
Socio-demographic factors									
*Age*									
15–24	1.00			1.00			1.00		
25–34	0.89		[0.59–1.32]	1.05		[0.73–1.53]	1.29		[0.53–3.11]
35–54	1.05		[0.66–1.69]	0.38	***	[0.24–0.59]	1.24		[0.50–3.08]
*Marital status*									
Never married	1.00						1.00		
Currently married	0.56	*	[0.34–0.92]	1.65	*	[1.01–2.69]	3.46	*	[1.17–10.28]
Formerly married	0.34	***	[0.19–0.59]	0.68		[0.39–1.18]	1.56		[0.48–5.05]
*Ethnicity*									
Kikuyu	1.00			1.00			1.00		
Luhya	1.57		[0.99–2.50]	1.28		[0.82–2.00]	0.98		0.46–2.11]
Luo	1.50		[0.93–2.42]	1.35		[0.86–2.12]	0.55		[0.22–1.38]
Kamba	0.86		[0.55–1.34]	0.92		[0.62–1.34]	0.33	**	[0.14–0.74]
Other	0.86		[0.55–1.35]	0.60	*	[0.39–0.93]	0.39	*	[0.17–0.89]
*Parity*									
No children	1.00			1.00			1.00		
1–2 children	0.68		[0.43–1.09]	2.84	***	[1.64–4.89]	2.70		[0.77–9.44]
3+ years	0.35	***	[0.19–0.65]	1.96	*	[1.03–3.3.74]	2.40		[0.67–8.69]
*Household size*									
1–3 members	1.00			1.00			1.00		
4–6 members	2.21	***	[1.52–3.23]	1.40	*	[1.01–1.94]	3.09	**	[1.45–6.58]
7+ members	2.03	**	[1.31–3.16]	1.22		[0.81–1.84]	2.72	*	[1.06–6.96]
Socio–economic factors									
*Education*									
None	1.00			1.00			1.00		
Primary	1.67	**	[1.17–2.38]	1.26		[0.93–1.71]	1.02		[0.52–1.99]
Secondary/Higher	2.07	*	[1.09–3.92]	1.79	*	[1.00–3.20]	2.27	*	[1.11–4.63]
*Wealth index*									
Poor	1.00			1.00			1.00		
Medium	0.90		[0.59–1.35]	0.79		[0.57–1.11]	0.64		[0.32–1.26]
Rich	0.81		[0.54–1.20]	0.69	*	[0.49–0.97]	0.39	**	m
*Residence*									
Slum	1.00			1.00			1.00		
Non-slum	15.33	***	[9.55–24.62]	3.61	***	[2.31–5.62]	10.03	***	[5.64–17.82]
*Employment status*									
Self-employed	1.00			1.00			1.00		
Informal	1.14		[0.66–1.98]	0.98		[0.63–1.52]	0.81		[0.31–2.08]
Formal	0.95		[0.55–1.65]	0.86		[0.51–1.43]	1.22		[0.54–2.76]
Unemployed	1.59	*	[1.05–2.40]	0.95		[0.69–1.32]	0.62		[0.31–1.25]
Behavioral/attitudinal factors									
*Pregnancy wantedness*									
Never pregnant	1.00			1.00			1.00		
Intended	0.25	***	[0.18–0.34]	2.30	***	[1.60–3.30]	6.48	***	[2.24–18.74]
Unintended	0.41	***	[0.27–0.62]	2.49	***	[1.61–3.84]	7.87	***	[2.51–24.70]

^*^ *p* < .05; **p < .01; ***p < .001

In Model II we controlled for socio-economic factors and women with secondary or higher education were more likely to use a traditional, short-term or long-term method than use no method compared to those with no education. Women from rich households were less likely to report use of short-term or long-term methods compared to using no method than those from poor households. As expected, women living in the non-slum settlements were more likely to use a traditional, short-term or long-term method than use no method compared to their counterparts living in the slum settlements. Considering employment status, unemployed women were more likely to use a traditional method than use no method compared to those who were self-employed. Model III controlled for behavioural/attitudinal factor, pregnancy wantedness, where women who reported that their pregnancy was intended and unintended were less likely to use a traditional method than use no method compared to those who had never been pregnant. On the contrary, these women were more likely to report use of a short-term or long-term method than use no method compared to those who were never pregnant.

## Discussion

Fewer contraceptive method choice studies make inter or intra-regional comparisons. Most studies focus on national or regional level data such as the DHS that allow contrasts at rural–urban level. The current study makes a contribution by broadening understanding of factors and determinants of contraceptive choice within an urban area while contrasting contraceptive behaviors among women living in slum and middle class non-slum settlements. Overall, the prevalence of contraceptive method choice was at 34.2 % for traditional methods, 31.2 % for short-term methods and only 6.4 % for long-term methods. 28.2 % of the women who were sexually active were not using any form of contraception. The 2008–09 Kenya DHS report use of any modern method among women aged 15–49 years as 53.6 %, additionally; use of any modern method among currently married women is reported at 53.1 % and 43.1 % for urban and rural women respectively [15]. The 2014 Kenya DHS report that the use of modern methods increased over the last decade from 32 – 53 % [17]. Comparison with national level data show much lower use in either urban settlement type which confirms the need to understand contraceptive use between and within regions.

Our results show that women who reported having at least one child were less likely to use traditional methods but more likely to use short-term or long-term methods. Further, our results show that the likelihood to use a long-term method increased with the number of children. This is an indication of the influence of number of children ever born on the choice of contraceptive method to adopt. Elsewhere, contraceptive use has been found to increase with parity, where women who had achieved their desired family size used contraceptives to limit births [35]. Women with three or more three children were more likely to use long term methods but less likely to use traditional or short-term methods compared to those with fewer children. Number of surviving children is a key determining factor in contraceptive use. Women who achieve the desired family size are therefore more likely to use long-term methods of contraception. According to the Kenya DHS survey, the reported ideal family size was 4 children and our results are a possible indication that women are more inclined towards that family size [15, 36]. Additionally, with the decrease in child mortality, more women are likely to use long-term contraception since they do not anticipate the need to replace a child [37]. On the other hand, women who had never been pregnant were using no methods; a finding similar to those from studies in western Europe [38, 39].

Further, our results suggest that older women were less likely to use traditional and short-term methods than those under 25 years but more likely to use long-term methods. This could be a possible indication that older women want to stop childbearing and are therefore more likely to use long-term methods which are more effective as opposed to younger women who want to use contraception to space hence more likely to use reversible or short-term methods [37, 40]. Although young women are increasingly initiating sex early, they are more disadvantaged in terms on contraceptive use as they receive no sex and contraceptive education [41].

As expected, there is a greater risk to experiencing pregnancy for women in marriage which explains their higher likelihood to use either short-term or long-term methods of contraception. Currently married women were more likely to use short-term and long-term methods of contraception compared to their never/formerly married counterparts. These findings largely confirm those of studies conducted in the Philippines and the US which found contraceptive use to be common in consistent relationships [40, 42].

Considering pregnancy wantedness, women who had reported intended and unintended pregnancy were less likely to use a traditional method, but more likely to use a short-term or long-term method. The likelihood to use either short-term or long-term method was higher for women who had reported unintended pregnancy. This could be an indication that women reporting intended pregnancies were not using any contraceptives for the pregnancy reported and therefore having the child at the right time. On the other hand, women who report unintended pregnancy made reference to the difficulties while carrying an accidental pregnancy and reported seeking appropriate contraceptive method soon after delivery [43]. Women living in non-slum settlements were more likely to use traditional methods, short-term or long-term methods than their counterparts living in slum. Slum settlements in Nairobi have been reported to be hubs of deprivation and risky health behaviours [20, 34].

Women working outside the home or those in formal employment were more likely to use contraception than those in self-employment. The increased likelihood to use traditional and long-term methods is partly attributed to the cost and benefit of child bearing and rearing. As is already documented elsewhere, childbearing and rearing is incompatible with employment outside the home. Additionally, engagement in productive employment increases women’s bargaining power which may result to higher contraceptive uptake [44, 45]. Women from rich households were less likely to use long-term methods. Similarly, Bangladesh women from rich households were found to be less likely to use permanent/long-term methods like sterilization for fear of the side effects or their mode of operation.

## Conclusions

The findings from this study suggest low use of both short-term and long-term methods among our study population. Majority of women reported use of traditional or no method with a few using short-term methods, and even fewer using long-term methods known to be more effective in pregnancy prevention. It is a fact that long-term methods require a doctor’s intervention for insertion and removal and many women may find this problematic especially when they come from resource constrained settings like slum settlements where access to such services may be problematic. Additionally, method choice is an indication of a successful family planning program. It is therefore important for the government to invest in increasing access of various contraceptive options by increasing the number of government health facilities thereby ultimately leading to increase contraceptive prevalence. More couples should be encouraged to take up contraception, and the process should include provision of a wide range of services to serve the diverse needs of these couples in the long-run. More women could benefit from additional awareness and education to dispel any myths and misconceptions around contraceptive use thereby addressing the benefits of long-term methods of contraception and ultimately increase overall contraceptive uptake. One major limitation to this study is that these are self-reports of the study participants. This study points to the need to address barriers to access of contraceptive options to allow women to make informed decisions on the methods that will be more appropriate to them based on their needs.

## Abbreviations

APHRC, african population and health research center; DHS, demographic and health survey; ICPD, international conference on population and development; LAM, lactational amenorrhea method; MDGs, millennium development goals; NUHDSS, Nairobi urban health and demographic surveillance system; OR, odds ratio; STEP UP, strengthening evidence for programming on unintended pregnancy.
